# A Modified 2 Tier Chemotherapy Response Score (CRS) and Other Histopathologic Features for Predicting Outcomes of Patients with Advanced Extrauterine High-Grade Serous Carcinoma after Neoadjuvant Chemotherapy

**DOI:** 10.3390/cancers13040704

**Published:** 2021-02-09

**Authors:** Yanping Zhong, Jinsong Liu, Xiaoran Li, Shannon N. Westin, Anais Malpica, Barrett C. Lawson, Sanghoon Lee, Bryan M. Fellman, Robert L. Coleman, Anil K. Sood, Nicole D. Fleming

**Affiliations:** 1 Department of Pathology, The University of Texas MD Anderson Cancer Center, Houston, TX 77030, USA; zhongyp@jlu.edu.cn (Y.Z.); XLi29@mdanderson.org (X.L.); amalpica@mdanderson.org (A.M.); BCLawson@mdanderson.org (B.C.L.); 2 Department of Pathology, The First Hospital of Jilin University, Changchun 130021, Jilin, China; 3 Gynecologic Oncology and Reproductive Medicine, The University of Texas MD Anderson Cancer Center, Houston, TX 77030, USA; swestin@mdanderson.org (S.N.W.); Robert.Coleman@usoncology.com (R.L.C.); asood@mdanderson.org (A.K.S.); NFleming@mdanderson.org (N.D.F.); 4 Systems Biology, The University of Texas MD Anderson Cancer Center, Houston, TX 77030, USA; SLee29@mdanderson.org; 5 Biostatistics, The University of Texas MD Anderson Cancer Center, Houston, TX 77030, USA; BMFellman@mdanderson.org

**Keywords:** extrauterine, high-grade serous carcinoma, neoadjuvant chemotherapy, chemotherapy response score, pathology features, survival

## Abstract

**Simple Summary:**

Reliable predictive indicators of response to neoadjuvant chemotherapy of advanced-stage extrauterine high-grade serous carcinoma are still lacking. Moreover, changes evident in tumor samples from interval surgery are not well understood. Our retrospective study aimed to address the prognostic value of chemotherapy response score (CRS) and identify additional predictive features for the risk of progression and death. In a cohort of 245 patients, we demonstrate that the modified two-tier CRS was prognostic for survival, and this significance was independent of scoring site; our data support expansion of CRS use to the adnexal samples. In addition to the CRS, oncocytic change, inflammation, and desmoplasia were additional histopathologic parameters related to survival. We recommend using the two-tier CRS, together with the additional histological features serving as secondary criteria for scoring, to identify patients at high risk for recurrence, allow tailored adjuvant therapy strategies, or consider clinical trials.

**Abstract:**

*Background*: The impact of chemotherapy response score (CRS) on prognosis has varied among studies. We addressed the prognostic significance of CRS and the prognostic value of previously undescribed histologic features using a cohort of 245 patients. *Methods*: Retrospective study in patients with advanced extrauterine high-grade serous carcinomas treated with neoadjuvant chemotherapy followed by interval tumor reductive surgery from 1990 to 2018 in our hospital. Gynecologic pathologists assessed tumor CRS and other histologic features. Clinical information was collected, and multivariate analyses were conducted. *Results*: A modified 2 tier CRS (CRS 1/2 versus CRS 3) was significantly associated, independent of scoring site (omental versus adnexal), with overall survival (OS) (omentum, *p* = 0.018; adnexa, *p* = 0.042; entire cohort, *p* = 0.002) and progression-free survival (PFS) (*p* = 0.021, *p* = 0.035, and *p* = 0.001, respectively). On multivariate survival analysis, 2 tier CRS, oncocytic change, inflammation, and desmoplasia were significant for OS (*p* = 0.034, *p* = 0.020, *p* = 0.007, and *p* = 0.010, respectively). Likewise, 2 tier CRS, inflammation, and desmoplasia were significant for PFS (*p* = 0.012, *p* = 0.003, *p* = 0.011, respectively). *Conclusions*: The modified 2 tier CRS was significantly associated with survival, independent of scoring site. Additional histologic features including oncocytic change, inflammation, and desmoplasia can also predict patient outcomes.

## 1. Introduction

Advanced extrauterine high-grade serous carcinoma (HGSC), including stage III and IV ovarian, fallopian tube, and peritoneal disease, is the most commonly diagnosed and the deadliest subtype of extrauterine carcinoma [1,2].

Neoadjuvant chemotherapy (NACT), followed by interval tumor reductive surgery (TRS), is increasingly used for patients with advanced extrauterine HGSC since upfront complete gross resection is not feasible for many patients. However, reliable predictive indicators of response to NACT are still lacking. Tumors can become difficult to recognize visually after NACT, and as a result, the assessment of residual disease via intraoperative evaluation or radiographic imaging may be inaccurate. Moreover, changes evident in tumor samples from interval surgery are not well understood, but histologic grading could be more reliable than clinical and radiologic evaluations for assessing response to treatment and even for identifying predictive features for the risk of progression and death [3,4].

Histologically, features of chemotherapy-related changes are regarded as indicators of regression in the tumors. Based on such post-treatment changes, chemotherapy response score (CRS) systems have been developed for histopathologic analysis in breast, esophageal, gastric, and rectal cancer and play an important role in guiding treatment and predicting prognosis [5,6].

Recently, the International Collaboration on Cancer Reporting (ICCR) recommended and the College of American Pathologists (CAP) endorsed a 3 tier CRS system for assessing cases of advanced extrauterine HGSC after NACT [7]. This tool is being used in some institutions but has yet to be accepted as routine clinical practice. The CRS mainly focuses on fibroinflammatory changes, pattern of invasion, and size of the largest focus of residual tumor. Other histologic features associated with chemotherapy-related changes are not included in the system, and whether those histologic features are clinically relevant has not been studied. In addition, the prognostic significance of the CRS system is still controversial. The CRS of omental tumor samples has been reported to correlate with progression-free survival (PFS) [3,4,7,8,9,10,11,12,13,14], but its predictive value for overall survival (OS) has differed among studies [3,4,7,8,9,10,11,12]. In addition, a wide range of conflicting results have been seen for the predictive value of CRS in adnexal tumor samples [10,12,13,14]. Therefore, the clinical usefulness of this CRS system remains to be further examined in a larger clinical cohort. To address this unmet need, we retrospectively examined a large cohort of patients with advanced extrauterine HGSC who were treated with NACT and interval TRS to define the clinical utility of omental CRS, adnexal CRS, and the full cohort CRS; and the extent to which other histologic features correlate with the CRS, OS, and PFS.

## 2. Materials and Methods

### 2.1. Study Population 

**Figure 1:**
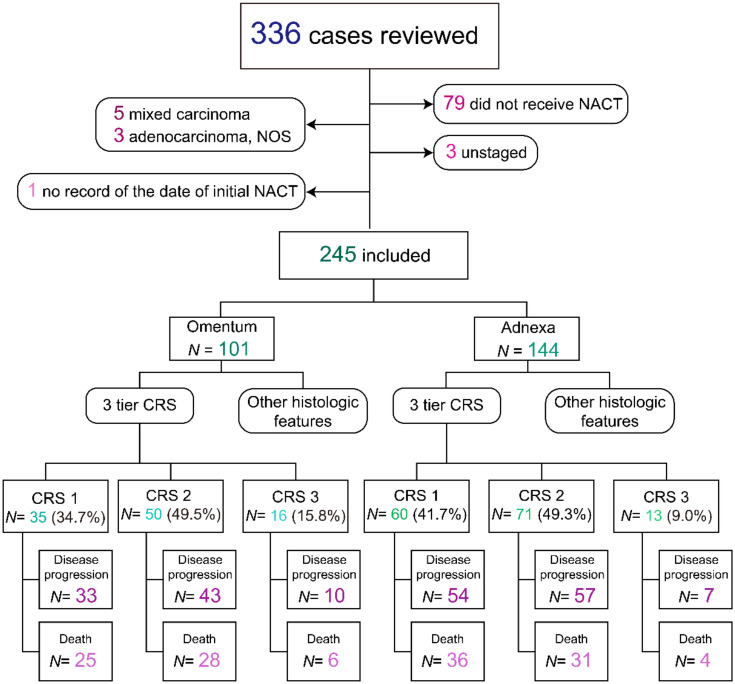
Overview of the study cohort. Of 336 patients with extrauterine carcinoma, 91 patients were excluded because patients did not receive neoadjuvant chemotherapy (NACT) (*N* = 79), tumor histology was not high-grade serous carcinoma (HGSC) (*N* = 8), staging information was not available (*N* = 3), or the date of initial NACT was unknown (*N* = 1). We included the remaining 245 patients in our analysis. Chemotherapy response score, CRS.

Study data were obtained after approval from the Institutional Review Board (IRB). Figure 1 provides an overview of the study cohort. The inclusion criteria were (1) primary ovarian, tubal, or peritoneal cancer; (2) pathologically confirmed HGSC; (3) International Federation of Gynecology and Obstetrics (FIGO) advanced stage (i.e., stage III–IV); (4) status of completed NACT and interval TRS. A retrospective review of histologic material was conducted which yielded 245 eligible cases out of 336 patients with advanced extrauterine HGSC treated at the University of Texas MD Anderson Cancer Center during a 28 year period (January 1990 to December 2018). In addition, BRCA status, follow-up duration, and status were recorded through electronic health record review. 

In most cases, NACT was given based on a routine regimen of a combination of platinum and taxane at our institution. Interval TRS included at minimum hysterectomy, salpingo-oophorectomy, and omentectomy.

### 2.2. Pathology Review

All available hematoxylin and eosin (H&E) slides from interval TRS specimens were reviewed by the study gynecologic pathologists, who were blinded to clinical outcomes.

**Figure 2:**
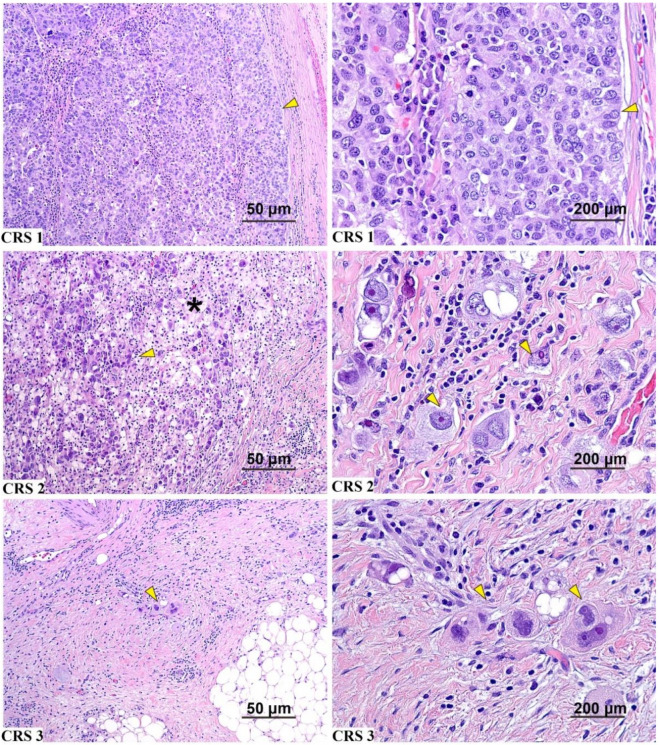
Three-tier CRS. Examples of the 3 tier CRS levels on H&E-stained slides from interval tumor reductive surgery (TRS) specimens (scale bars: left column, 50 μm; right column, 200 μm). CRS 1: no or minimal tumor response; CRS 2: appreciable tumor response amid viable tumor that is readily identifiable; CRS 3: complete or near-complete response with no residual tumor or minimal irregularly scattered tumor foci seen as individual cells. Yellow arrows show tumor areas and the asterisk show infiltration of foamy histiocytes.

The 3 tier CRS system, described by Böhm et al. [3], was used to assign a score for the chemotherapy response. Chemotherapy response score 1 is defined as no or minimal tumor response that is limited to a few foci or cases in which it is difficult to determine response or tumor-associated desmoplasia. Chemotherapy response score 2 is defined as appreciable tumor response amidst viable tumor with both readily identifiable and regularly distributed tumor. Chemotherapy response score 3 is defined as complete or near complete response with no residual tumor or minimal irregularly scattered tumor foci as individual cells or cell groups measuring a maximum of 2 mm each (Figure 2). The CRS score is assigned based on the area of least response. For the purpose of this study, the CRS was ultimately applied to the omentum or adnexa. Cases with the same grading score for both sites are assigned by a larger sample or more massive tumor. Chemotherapy response scores were compared in two ways: the 3 tier system (CRS 1 versus 2 versus 3) and a modified 2 tier system (CRS 1/2 versus 3). 

**Figure 3:**
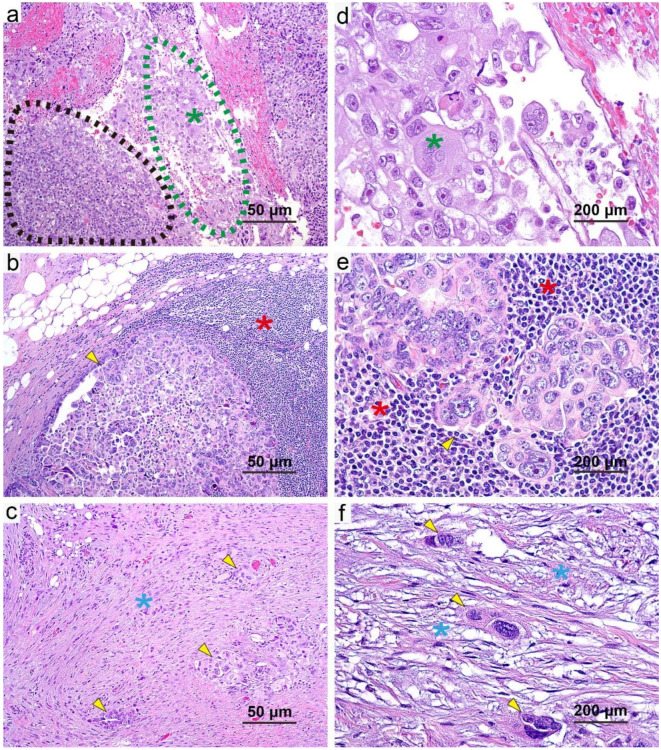
Representative examples of the histologic features that predicted survival in our cohort (H&E staining; scale bars: (**a**–**c**) 50 μm, (**d**–**f**) 200 μm). (**a**,**d**) Oncocytic changes (green, dotted line area with green asterisks): the abundant eosinophilic cytoplasm gives those tumor cells a pink appearance in contrast to adjacent other tumor cells (black, dotted line area). (**b**,**e**) Inflammation (red asterisks): inflammatory cells infiltrate around the tumor nest. (**c**,**f**) Desmoplasia (blue asterisks): proliferative fibroblasts with myxoid stroma are located at the periphery of the tumor foci. The yellow arrows show tumor areas.

**Figure 4:**
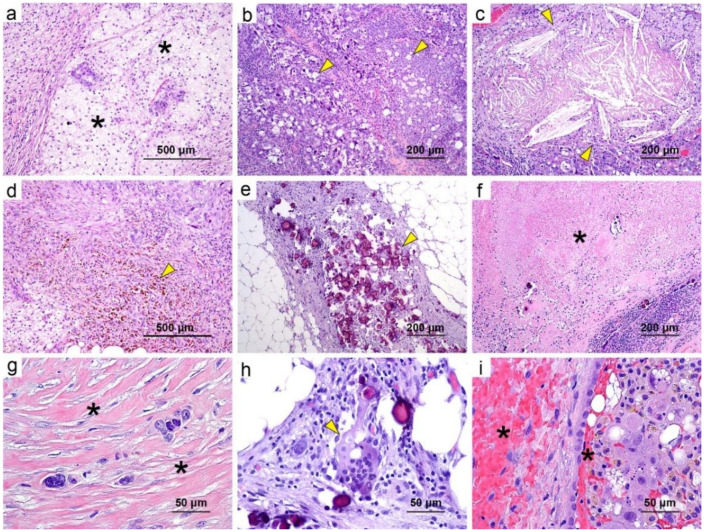
Representative examples of the histologic features that were not significantly associated with survival in our cohort (H&E staining; scale bars: (**a**,**d**) 500 μm, (**b**,**c**,**e**,**f**) 200 μm, (**g**–**i**) 50 μm). (**a**) Foamy histiocytes (black asterisks), score: 3; (**b**) eosinophilic cytoplasm with vacuolization (yellow arrows), score: 2; (**c**) cholesterol crystal (yellow arrow), score: 2; (**d**) hemosiderin deposition (yellow arrow), score: 2; (**e**) calcification/psammoma bodies (yellow arrow), score: 2; (**f**) necrosis (black asterisk), score: 2; (**g**) scarry fibrosis (black asterisks), score: 2; (**h**) foreign body giant cells (yellow arrow), score: 1; (**i**) hemorrhage (black asterisks), score: 1.

**Figure 5:**
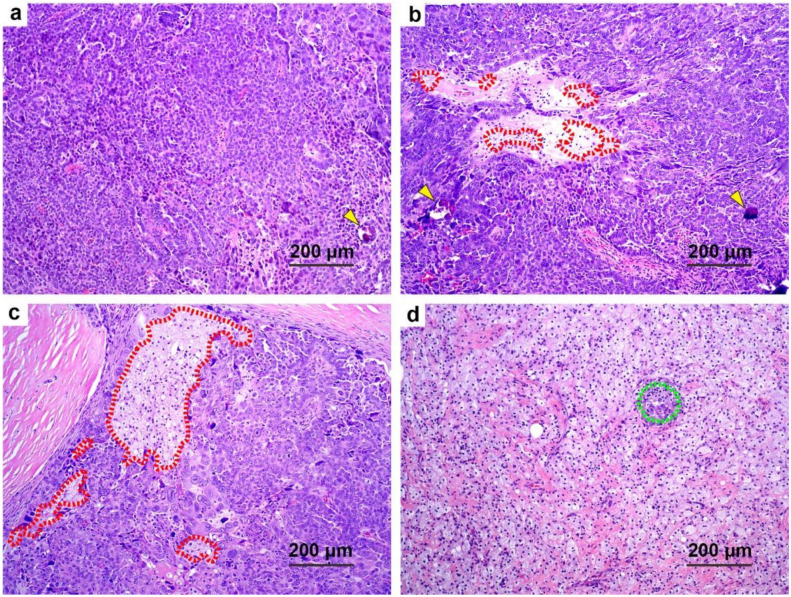
A representative example illustrates the scoring criteria of additional histopathologic features associated with treatment. Different grade foamy histiocytes response to neoadjuvant chemotherapy (H&E staining; scale bars: 200 μm). (**a**) 0: No foamy histiocytes response; (**b**) 1: foamy histiocytes < 5% (areas of red, dotted line); (**c**) 2: foamy histiocytes 5–50% (areas of red, dotted line); (**d**) 3: foamy histiocytes > 50%, massive foamy histiocytes infiltrate around a tumor cell nest (the area of green, dotted line). Yellow arrows indicate psammoma bodies (**a**,**b**).

We also recorded other histologic features, including tumor cytopathologic changes and alterations of both tumoral and non-tumoral stroma. Supplementary Materials Table S1 lists the features we assessed for, with representative examples shown in Figure 3 and Figure 4. Cytopathologic changes observed were eosinophilic cytoplasm with vacuolization, oncocytic change, and necrosis. Histologic features of the stroma included infiltrating of foamy histiocytes, inflammation, desmoplasia, scarry fibrosis, calcification/psammoma bodies, hemosiderin deposition, foreign-body giant cells, cholesterol crystals, and hemorrhage. A semi-quantitative score (a representative example is shown in Figure 5) was used for the histologic features not included in the CRS: 0, no presence; 1, minimal presence (<5%); 2, focal occurrence (5–50%); 3, widespread occurrence (>50%). Histologic features were compared in two ways (0/1 versus 2/3 and 0/1 versus 2 versus 3). 

For 40 randomly selected cases from the study cohort, the CRS and other histologic features were scored by four gynecologic pathologists (Y.Z., J.L., A.M., B.C.L.) independently. Then, the results were discussed and consensus reached using a multi-headed microscope. Two of the four pathologists (Y.Z. and J.L.) scored the remaining cases following the consensus.

### 2.3. Data Analyses and Statistical Methods

Summary statistics, such as means, standard deviations, ranges, frequencies, and percentages, were used to describe the study population. Overall survival and PFS were estimated using the Kaplan–Meier product-limit estimator and modeled via Cox proportional hazards regression. Overall survival was measured from the date of the initial NACT cycle to date of death or last follow-up. Progression-free survival was measured from the date of the initial NACT cycle to the date of the last clinic visit, date of first recurrence or progression, or date of death. Patients’ progression-free and alive statuses were censored at the date of last clinic visit. Univariate and multivariable analyses for PFS and OS were performed based on the entire cohort. A final multivariable model was constructed using backwards selection where the original model included all variables significant (*p* < 0.05) in the univariate setting. Exploratory associations of OS and PFS with pathology data were conducted accordingly. No adjustments were made for multiple testing. Histologic features scores were summarized by CRS and compared using Fisher’s exact test. Statistical significance was designated as a *p*-value less than 0.05, and the 95% confidence intervals (CIs) for hazard rate ratios were calculated. All statistical analyses were performed using Stata/MP v16.0 (College Station, TX, USA).

## 3. Results

### 3.1. Baseline Characteristics

Table 1 summarizes the clinical findings of the 245 patients who were eligible for analysis. 

The median age was 62 years (range, 29–81 years). For 234 (95.5%) cases, carboplatin, and paclitaxel formed the basis of NACT. Among these, a total of 217 (88.6%) patients were treated with only neoadjuvant carboplatin/paclitaxel, and 17 cases were treated with carboplatin/paclitaxel plus additional agents: docetaxel in nine cases, bevacizumab in four cases, bevacizumab + gemcitabine in one case, cisplatin + gemcitabine in one case, liposomal doxorubicin in one case, and bevacizumab + cyclophosphamide in one case. In 11 cases, treatment was platinum based (without paclitaxel): carboplatin only (one case), carboplatin combined with additional agents (docetaxel in five cases, gemcitabine in one case, and cyclophosphamide in one case), cisplatin combined with additional agents (cyclophosphamide in one case and cyclophosphamide + doxorubicin in one case), or carboplatin and cisplatin combined with docetaxel (one case). There were 168 (68.6%) cases of 3–4 cycles of NACT, 75 (30.6%) cases of more than four cycles, and two (0.8%) cases were unknown.

### 3.2. Follow-Up, Survival, and Observed Event Rate

The median follow-up was 27 months (range, 5–160 months). The median OS and median PFS were 41 months (95% CI: 36–48 months) and 13 months (95% CI: 12–14 months), respectively. At the time of data cutoff, PFS events (progression or recurrence) had occurred in 83.3% (204 of 245) of patients and OS events (death) in 53.1% (130 of 245) (Figure 1).

### 3.3. BRCA Mutation Status and Survival

Germline BRCA (gBRCA) mutation information was available for 127 cases in the study cohort: 28 (22%) were gBRCA positive and 99 (78%) were gBRCA negative. Germline BRCA-positive status was associated with longer PFS (*p* = 0.021; Supplementary Materials Figure S1a). The median PFS for the gBRCA-positive group was 19 months versus 12 months for the gBRCA-negative group. When gBRCA status was correlated to OS, the gBRCA-positive group also appeared to have improved OS, although this finding did not reach significance (median OS: 62 months for the gBRCA-positive group versus 51 months for the gBRCA-negative group; *p* = 0.137, Supplementary Materials Figure S1b).

### 3.4. Correlation of Additional Histologic Features with CRS 

The scores for the 3 tier CRS and additional 12 histologic features described in the material and methods assessed in this study are shown in Supplementary Materials Table S1. We grouped these 12 histologic feature scores of 0/1 versus 2/3 and found the following five features were related to CRS: eosinophilic cytoplasm with vacuolization, oncocytic change, foamy histiocytes, desmoplasia, and necrosis (*p* = 0.021, *p* = 0.020, *p* < 0.001, *p* = 0.025, and *p* = 0.033, respectively; Supplementary Materials Table S2). For the percentages of scores of 2/3, there appeared to be gradual increases in distribution for foamy histiocytes with increasing CRS (35.8%, 59.5%, and 69.0% for CRS 1, 2, and 3, respectively; *p* < 0.001) and decreases for desmoplasia (65.3%, 62.8%, and 37.9% for CRS 1, 2, and 3, respectively; *p* = 0.025) and necrosis (24.2%, 19.8%, and 3.5% for CRS 1, 2, and 3, respectively; *p* = 0.033). No significant correlation was seen between CRS and remaining histologic features. 

When compared histologic feature scores by 0/1 versus 2 versus 3, we obtained approximately the same results with a less significant gradual increase or decrease in distribution. The same features were significantly correlated with CRS, with the addition of scarry fibrosis (*p* = 0.043).

### 3.5. 2 Tier CRS Showed Stronger Prognostic Significance

**Figure 6:**
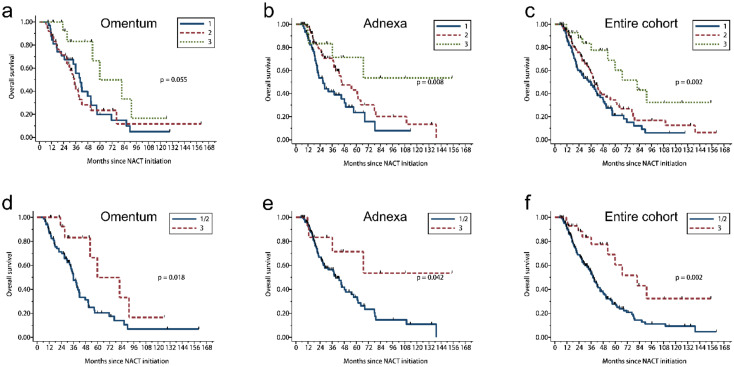
Kaplan–Meier estimates of OS according to CRS for the entire cohort and scoring site subgroups. (**a**–**c**) Three-tier CRS. (**d**–**f**) Modified 2 tier CRS (CRS 1 and 2 versus CRS 3).

**Figure 7:**
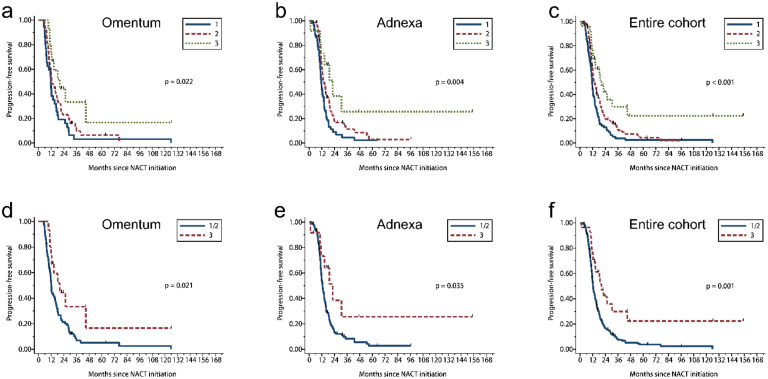
Kaplan–Meier estimates of PFS according to CRS for the entire cohort and scoring site subgroups. (**a**–**c**) Three-tier CRS. (**d**–**f**) Modified 2 tier CRS (CRS 1 and 2 versus CRS 3).

We analyzed CRS by the site of the tissue sample of least response, larger tumor or sample (when same grading score for both sites): omentum (*n* = 101) or adnexa (*n* = 144) and for the cohort as a whole (omental + adnexal, *n* = 245). Chemotherapy response score distribution for these groups is shown in Supplementary Materials Figure S2. In the omental group, CRS 1 was observed in 35 (34.7%) cases; CRS 2, 50 (49.5%) cases; and CRS 3, 16 (15.8%) cases. The omental CRS was significantly associated with OS in the 2 tier comparison (CRS 1/2 versus 3; *p* = 0.018, Table 2, Figure 6d) but not in the 3 tier comparison (CRS 1 versus 2 versus 3; *p* = 0.055, Table 2, Figure 6a). The omental score was significantly associated with PFS, for both the 3 tier and 2 tier CRS systems (*p* = 0.022 and *p* = 0.021, respectively; Table 2, Figure 7a,d). 

In the adnexal group, CRS 1 was observed in 60 (41.7%) cases; CRS 2, 71 (49.3%) cases; CRS 3, 13 (9.0%) cases. Using both the 3 tier and 2 tier systems, CRS was significantly associated with OS (*p* = 0.008 and *p* = 0.042, respectively; Table 2, Figure 6b,e) and PFS (*p* = 0.004 and *p* = 0.035, respectively; Table 2, Figure 7b,e). 

In the entire cohort with both omentum and adnexa, CRS 1 was observed in 95 (38.8%); CRS 2, 121 (49.4%) cases; CRS 3, 29 (11.8%) cases. CRS was significantly associated with OS (*p* = 0.002 and *p* = 0.002, respectively; Table 2, Figure 6c,f) and PFS (*p* < 0.001 and *p* = 0.001, respectively; Table 2, Figure 7c,f) using either 3 tier or 2 tier systems. Taken together, the 2 tier CRS system was associated with OS and PFS for the omental group, adnexal group, or both CRS; while the 3 tier CRS system failed to predict OS in omentum.

### 3.6. Histologic Features and Patient Survival in Univariate Analysis

To determine if other histologic features other than CRS can predict survival outcomes, we performed univariate analysis between the 12 histologic features and survival and identified the histologic variables that were significantly correlated with PFS and OS (Supplementary Materials Table S3). With histologic feature scores grouped as 0/1 versus 2/3, inflammation, oncocytic change, desmoplasia, and foamy histiocytes showed significant correlation with OS (*p* = 0.011, *p* = 0.009, *p* = 0.006, and *p* = 0.047, respectively). Inflammation, desmoplasia, foamy histiocytes, and foreign body giant cells showed significant correlation with PFS (*p* = 0.010, *p* = 0.009, *p* = 0.038, and *p* = 0.008, respectively). With histologic feature scores compared between 0/1 versus 2 versus 3, similar results were obtained between these variables and survival but accompanied by less significant trends.

### 3.7. Multivariate Model of Survival Based on CRS and New Histopathologic Features

To determine if these identified new histologic features can further stratify chemotherapy response in addition to CRS, we conducted a multivariate analysis for survival and obtained the multivariate OS and PFS models for the entire cohort (Table 3). The representative images of prognostic histologic features of oncocytic change, inflammation, and desmoplasia are shown in Figure 3. Variables associated with OS in the final model were 2 tier CRS, oncocytic change, inflammation, and desmoplasia. Higher scores for CRS (CRS 3 versus CRS 1/2, HR: 0.49; 95% CI: 0.25–0.95; *p* = 0.034), oncocytic change (0/1 versus 2/3, HR: 0.63; 95% CI: 0.42–0.93; *p* = 0.020), and inflammation (0/1 versus 2/3, HR: 0.60; 95% CI: 0.41–0.87; *p* = 0.007) were associated with better OS. A higher score for desmoplasia was associated with worse OS (0/1 versus 2/3, HR: 1.67; 95% CI: 1.13–2.47; *p* = 0.010).

Variables associated with PFS in the final model were 2 tier CRS, inflammation, and desmoplasia. Higher scores for CRS (CRS 3 versus CRS 1/2, HR: 0.52; 95% CI: 0.31–0.87; *p* = 0.012) and inflammation (0/1 versus 2/3, HR: 0.63; 95% CI: 0.46–0.85; *p* = 0.003) were associated with better PFS, whereas a higher score for desmoplasia was associated with worse PFS (0/1 versus 2/3, HR: 1.48; 95% CI: 1.09–2.01; *p* = 0.011). 

## 4. Discussion

The main finding from this study was that the 2 tier CRS exhibited a strong association with survival after NACT and interval TRS that was independent of scoring site (omental versus adnexal). In addition, we identified histopathologic changes, such as oncocytic change, inflammation, and desmoplasia, that were prognostic factors for outcomes in multivariate analysis.

The 3 tier CRS system was proposed in 2015 and recently recommended by ICCR and CAP in ovarian cancer pathology reporting guidelines [3,7]. Subsequently, several studies have further examined this system and correlated it with prognosis [4,8,9,10,11,12,13,14]. However, these results showed variable prognostic value, particularly in scoring adnexal samples [8,10,12,13,14]. Most studies showed consistent results when using modified 2 tier CRS as compared to the 3 tier CRS in the predicting PFS based on omental tumor sample, while these studies yield conflicting results when using 2 tier or 3 tier CRS predicting OS or using adnexal tumor sample [4,8,9,10,11,12,14]. In our results, the modified 2 tier CRS strongly correlated with both OS and PFS, regardless of omental or adnexal origin, while the 3 tier CRS can similarly predict survival except in predicting OS from omental samples, suggesting that 2 tier may have better predictive value overall.

Discrepancies of the present study with previous ones could be due to the sample size or patient population. Our study represents the largest cohort reported from a single institution to date, and survival event (death for OS; progression for PFS) was observed for most cases, which allowed analyses based on mature data, especially for OS. This may explain why our data demonstrated significant correlation of CRS with OS. Compared with other studies, we had a small percentage of CRS 3 (11.8%) cases, which may be due to the relatively large percentage of adnexal cases (58.8%) in our cohort. It has been reported that CRS 3 is less common for adnexal CRS than for omental CRS [8,10]. Alternatively, it could be also due to the number of chemotherapy cycles in different cohorts.

Oncocytic change is described as tumor cells with abundant eosinophilic cytoplasm, which gives tumor cells a “pink” appearance on H&E-stained slides. Immunohistochemistry and electron microscopy have revealed that oncocytic changes are caused by the accumulation of large numbers of mitochondria in the cell [15,16,17]. We found that oncocytic change was associated with improved OS. As a treatment response, such change may represent degeneration of tumor cells or a differentiation-inducing phenomenon triggered by treatment.

Desmoplasia is considered a cancer-associated extracellular feature, which is mainly composed of proliferative fibroblasts with myxoid stroma and located at the periphery of the tumor foci. It participates in forming the microenvironment and also involves affecting therapeutic efficacy via either constructing a mechanical barrier that blocks chemotherapy and host immunity or contributes to the anti-angiogenic effect [18,19]. Similar implications were observed in our results, which indicated desmoplasia was related to poor chemotherapy-response and worse prognosis.

Links between host immunity and survival have been revealed in multiple studies [20,21]. Tumor-infiltrating inflammatory cells are strongly associated with survival in carcinomas including ovarian cancer [22]. In the current study, the infiltrating inflammatory cells were dominantly lymphocytes and plasma cells, with a small number of neutrophils and eosinophils. Inflammation was predictive for survival in our findings, which raises the possibility of investigating tumor-specific immune response in HGSC after NACT with potential relevance to immunotherapy and targeted therapy.

The limitations of our study are that it was retrospective and from a single center. Therefore, the findings presented hereby still need further validation. The major strengths are as follows: we showed for the first time that histopathologic parameters combined with CRS had a prognostic implication on survival in patients with advanced extrauterine HGSC after NACT. Our data represent the largest single-institution cohort with sufficiently mature data.

## 5. Conclusions

We demonstrate that the 2 tier CRS was prognostic for both PFS and OS in advanced extrauterine HGSC after NACT, and this significance was independent of scoring site. Our data support expansion of CRS use to the adnexal samples, and the use of the 2 tier CRS should be considered over the 3 tier CRS system. In addition to the CRS, oncocytic change, inflammation, and desmoplasia were additional histopathologic parameters that were related to survival. The simplified scoring system with fewer tiers and identifiable cut-off values makes it easy to use and maximize reproducibility. We recommend that the additional histological features be incorporated into the CRS system serving as secondary criteria for scoring.

## Figures and Tables

**Table 1:** Baseline characteristics of the study cohort (*n* = 245).

Characteristics	*N* (%)
Age (years)	
Median (range)	62 (29–81)
Primary site	
Tubo-ovarian	216/245 (88.2)
Peritoneal	29/245 (11.8)
FIGO stage	
III	105/245 (42.9)
IV	125/245 (51.0)
Advanced, not otherwise specified	15/245 (6.1)
BRCA status (*n* = 127)	
BRCA positive	28/127 (22.0)
BRCA negative	99/127 (78.0)
Cycles of NACT	
3 or 4	168/245 (68.6)
>4	75/245 (30.6)
Unknown	2/245 (0.8)
Total of ≥6 cycles of chemotherapy	
Neoadjuvant+adjuvant	194/245 (79.2)
Regimen of NACT	
Carboplatin/paclitaxel only	217/245 (88.6)
Carboplatin/paclitaxel and additional agent(s)	17/245 (6.9)
Platinum-based therapy (no paclitaxel)	11/245 (4.5)
Scored site	
Omentum	101/245 (41.2)
Adnexa	144/245 (58.8)
Follow-up (months)	
Median (range)	27 (5–160)

**Table 2:** Survival analysis by CRS for each scored tissue sample site and the entire cohort.

Site	CRS	OS				PFS			
		*N*	Events	Median	*p*-Value *	*N*	Events	Median	*p*-Value *
Omentum	3 tier				0.055				0.022
	1	35	25	40.5		35	33	11.7	
	2	50	28	34.4		49	43	12.7	
	3	16	6	59.9		15	10	20.3	
	2 tier				0.018				0.021
	1/2	85	53	36.0		84	76	12.2	
	3	16	6	59.9		15	10	20.3	
Adnexa	3 tier				0.008				0.004
	1	60	36	26.9		60	54	11.5	
	2	71	31	45.2		71	57	14.0	
	3	13	4	NE		12	7	22.3	
	2 tier				0.042				0.035
	1/2	131	67	39.5		131	111	12.6	
	3	13	4	NE		12	7	22.3	
Entire cohort	3 tier				0.002				<0.001
	1	95	61	35.9		95	87	11.6	
	2	121	59	39.1		120	100	14.0	
	3	29	10	81.8		27	17	20.3	
	2 tier				0.002				0.001
	1/2	216	120	38.0		215	187	12.4	
	3	29	10	81.8		27	17	20.3	

* Log–rank test. Overall survival, OS; progression-free survival, PSF; not estimable, NE.

**Table 3:** Multivariate survival analysis by prognostic factor.

Survival	Histopathology	HR	95% CI		*p*-Value
			LB	UB	
OS	2 tier CRS (1/2 versus 3)	0.49	0.25	0.95	0.034
	Oncocytic change (0/1 versus 2/3)	0.63	0.42	0.93	0.020
	Inflammation (0/1 versus 2/3)	0.60	0.41	0.87	0.007
	Desmoplasia (0/1 versus 2/3)	1.67	1.13	2.47	0.010
PFS	2 tier CRS (1/2 versus 3)	0.52	0.31	0.87	0.012
	Inflammation (0/1 versus 2/3)	0.63	0.46	0.85	0.003
	Desmoplasia (0/1 versus 2/3)	1.48	1.09	2.01	0.011

Hazard ratio, HR; confidence interval, CI; lower bound, LB; upper bound, UB.

## Supplementary Materials

The following are available online at https://www.mdpi.com/2072-6694/13/4/704/s1. Figure S1: Kaplan–Meier estimates of survival according to BRCA mutation status. (a) BRCA mutation was associated with longer PFS and decreased risk of recurrence; (b) BRCA mutation was non-significantly associated with OS. Figure S2: Distribution of scores using the 3 tier CRS for the entire cohort and subgroups according to specimen site. Table S1: The CRS and other histologic feature scores for the entire cohort. Table S2: Identified histologic features with statistically significant associations with CRS. Table S3: Univariate survival analysis by prognostic factor.
