# Supplementary material for: A Modified 2 Tier Chemotherapy Response Score (CRS) and Other Histopathologic Features for Predicting Outcomes of Patients with Advanced Extrauterine High-Grade Serous Carcinoma after Neoadjuvant Chemotherapy
Source: Cancers (Basel). 2021 Feb 9;13(4):704. doi: 10.3390/cancers13040704 (PMC7916221; doi:10.3390/cancers13040704)
Supplement: Supplementary file 1 [file cancers-13-00704-s001.zip › Table S1.docx]

**Table S1**. The CRS and other histologic feature scores for the entire cohort

| **Histopathology** | ***N*** | **%** |
| --- | --- | --- |
|  |  |  |
| 3-tier CRS |  |  |
| 1 | 95 | 38.8 |
| 2 | 121 | 49.4 |
| 3 | 29 | 11.8 |
| Eosinophilic cytoplasm with vacuolization |  |  |
| 0 | 119 | 48.6 |
| 1 | 57 | 23.3 |
| 2 | 58 | 23.7 |
| 3 | 11 | 4.5 |
| Oncocytic change |  |  |
| 0 | 101 | 41.2 |
| 1 | 59 | 24.1 |
| 2 | 75 | 30.6 |
| 3 | 10 | 4.1 |
| Foamy histiocytes |  |  |
| 0 | 64 | 26.1 |
| 1 | 55 | 22.5 |
| 2 | 93 | 37.9 |
| 3 | 33 | 13.5 |
| Scarry fibrosis |  |  |
| 0 | 174 | 71.0 |
| 1 | 15 | 6.1 |
| 2 | 48 | 19.6 |
| 3 | 8 | 3.3 |
| Inflammation |  |  |
| 0 | 13 | 5.3 |
| 1 | 68 | 27.8 |
| 2 | 138 | 56.3 |
| 3 | 26 | 10.6 |
| Calcification/psammoma bodies |  |  |
| 0 | 45 | 18.4 |
| 1 | 100 | 40.8 |
| 2 | 78 | 31.8 |
| 3 | 22 | 9.0 |
| Hemosiderin deposition |  |  |
| 0 | 125 | 51.0 |
| 1 | 59 | 24.1 |
| 2 | 58 | 23.7 |
| 3 | 3 | 1.2 |
| Desmoplasia |  |  |
| 0 | 65 | 26.5 |
| 1 | 31 | 12.7 |
| 2 | 92 | 37.6 |
| 3 | 57 | 23.3 |
| Necrosis |  |  |
| 0 | 185 | 75.5 |
| 1 | 12 | 4.9 |
| 2 | 33 | 13.5 |
| 3 | 15 | 6.1 |
| Foreign-body giant cells |  |  |
| 0 | 213 | 86.9 |
| 1 | 23 | 9.4 |
| 2 | 9 | 3.7 |
| 3 | 0 | 0.0 |
| Cholesterol crystals |  |  |
| 0 | 194 | 79.2 |
| 1 | 25 | 10.2 |
| 2 | 25 | 10.2 |
| 3 | 1 | 0.4 |
| Hemorrhage |  |  |
| 0 | 214 | 87.4 |
| 1 | 13 | 5.3 |
| 2 | 17 | 6.9 |
| 3 | 1 | 0.4 |
