# Supplementary material for: A Modified 2 Tier Chemotherapy Response Score (CRS) and Other Histopathologic Features for Predicting Outcomes of Patients with Advanced Extrauterine High-Grade Serous Carcinoma after Neoadjuvant Chemotherapy
Source: Cancers (Basel). 2021 Feb 9;13(4):704. doi: 10.3390/cancers13040704 (PMC7916221; doi:10.3390/cancers13040704)
Supplement: Supplementary file 1 [file cancers-13-00704-s001.zip › Table S2.docx]

**Table S2.** Identified histologic features with statistically significant associations with CRS

| **Histopathologic feature** | **CRS 1** | | **CRS 2** | | **CRS 3** | | ***P*-value*** |
| --- | --- | --- | --- | --- | --- | --- | --- |
|  | ***N*** | **%** | ***N*** | **%** | ***N*** | **%** |  |
| Eosinophilic cytoplasm with vaculization | | |  |  |  |  | 0.021 |
| 0/1 | 71 | 74.7 | 79 | 65.3 | 26 | 89.7 |  |
| 2/3 | 24 | 25.3 | 42 | 34.7 | 3 | 10.3 |  |
| Oncocytic change |  |  |  |  |  |  | 0.020 |
| 0/1 | 68 | 71.6 | 69 | 57 | 23 | 79.3 |  |
| 2/3 | 27 | 28.4 | 52 | 43 | 6 | 20.7 |  |
| Foamy histiocytes |  |  |  |  |  |  | <0.001 |
| 0/1 | 61 | 64.2 | 49 | 40.5 | 9 | 31 |  |
| 2/3 | 34 | 35.8 | 72 | 59.5 | 20 | 69 |  |
| Desmoplasia |  |  |  |  |  |  | 0.025 |
| 0/1 | 33 | 34.7 | 45 | 37.2 | 18 | 62.1 |  |
| 2/3 | 62 | 65.3 | 76 | 62.8 | 11 | 37.9 |  |
| Necrosis |  |  |  |  |  |  | 0.033 |
| 0/1 | 72 | 75.8 | 97 | 80.2 | 28 | 96.6 |  |
| 2/3 | 23 | 24.2 | 24 | 19.8 | 1 | 3.5 |  |

*Histopathologic features were compared by CRS using chi-squared test or Fisher’s exact test when appropriate.
