# Supplementary material for: A Modified 2 Tier Chemotherapy Response Score (CRS) and Other Histopathologic Features for Predicting Outcomes of Patients with Advanced Extrauterine High-Grade Serous Carcinoma after Neoadjuvant Chemotherapy
Source: Cancers (Basel). 2021 Feb 9;13(4):704. doi: 10.3390/cancers13040704 (PMC7916221; doi:10.3390/cancers13040704)
Supplement: Supplementary file 1 [file cancers-13-00704-s001.zip › Table S3.docx]

**Table S3.** Univariate survival analysis by prognostic factor

| **Survival** | **Histopathology** | ***N*** | **Events** | **Median** | **log-rank** | **HR** | **95% LB** | **95% UB** | ***P*-value** |
| --- | --- | --- | --- | --- | --- | --- | --- | --- | --- |
|  |  |  |  |  |  |  |  |  |  |
| OS | 2-tier CRS |  |  |  | 0.002 |  |  |  |  |
|  | 1/2 | 216 | 120 | 38.0 |  | 1.00 | 1.00 | 1.00 |  |
|  | 3 | 29 | 10 | 81.8 |  | 0.38 | 0.20 | 0.73 | 0.004 |
|  | Inflammation |  |  |  | 0.011 |  |  |  |  |
|  | 0/1 | 81 | 51 | 28.6 |  | 1.00 | 1.00 | 1.00 |  |
|  | 2/3 | 164 | 79 | 42.1 |  | 0.63 | 0.45 | 0.90 | 0.012 |
|  | Oncocytic change |  |  |  | 0.009 |  |  |  |  |
|  | 0/1 | 160 | 95 | 36.0 |  | 1.00 | 1.00 | 1.00 |  |
|  | 2/3 | 85 | 35 | 45.2 |  | 0.60 | 0.41 | 0.88 | 0.010 |
|  | Desmoplasia |  |  |  | 0.006 |  |  |  |  |
|  | 0/1 | 96 | 43 | 52.6 |  | 1.00 | 1.00 | 1.00 |  |
|  | 2/3 | 149 | 87 | 37.7 |  | 1.67 | 1.15 | 2.41 | 0.006 |
|  | Foamy histocytes |  |  |  | 0.047 |  |  |  |  |
|  | 0/1 | 119 | 71 | 36.0 |  | 1.00 | 1.00 | 1.00 |  |
|  | 2/3 | 126 | 59 | 45.1 |  | 0.71 | 0.50 | 1.00 | 0.048 |
| PFS | 2-tier CRS |  |  |  | 0.001 |  |  |  |  |
|  | 1/2 | 215 | 187 | 12.4 |  | 1.00 | 1.00 | 1.00 |  |
|  | 3 | 27 | 17 | 20.3 |  | 0.44 | 0.27 | 0.73 | 0.002 |
|  | Inflammation |  |  |  | 0.010 |  |  |  |  |
|  | 0/1 | 81 | 72 | 11.9 |  | 1.00 | 1.00 | 1.00 |  |
|  | 2/3 | 161 | 132 | 13.3 |  | 0.68 | 0.51 | 0.91 | 0.010 |
|  | Desmoplasia |  |  |  | 0.009 |  |  |  |  |
|  | 0/1 | 95 | 73 | 14.7 |  | 1.00 | 1.00 | 1.00 | . |
|  | 2/3 | 147 | 131 | 12.0 |  | 1.46 | 1.10 | 1.95 | 0.010 |
|  | Foamy histocytes |  |  |  | 0.038 |  |  |  |  |
|  | 0/1 | 119 | 105 | 12.2 |  | 1.00 | 1.00 | 1.00 |  |
|  | 2/3 | 123 | 99 | 14.4 |  | 0.75 | 0.57 | 0.99 | 0.039 |
|  | Foreign-body giant cells | |  |  | 0.008 |  |  |  |  |
|  | 0/1 | 233 | 196 | 12.9 |  | 1.00 | 1.00 | 1.00 |  |
|  | 2/3 | 9 | 8 | 10.6 |  | 2.55 | 1.24 | 5.22 | 0.011 |
