# Supplementary figures and images for: A Modified 2 Tier Chemotherapy Response Score (CRS) and Other Histopathologic Features for Predicting Outcomes of Patients with Advanced Extrauterine High-Grade Serous Carcinoma after Neoadjuvant Chemotherapy
Source: Cancers (Basel). 2021 Feb 9;13(4):704. doi: 10.3390/cancers13040704 (PMC7916221; doi:10.3390/cancers13040704)

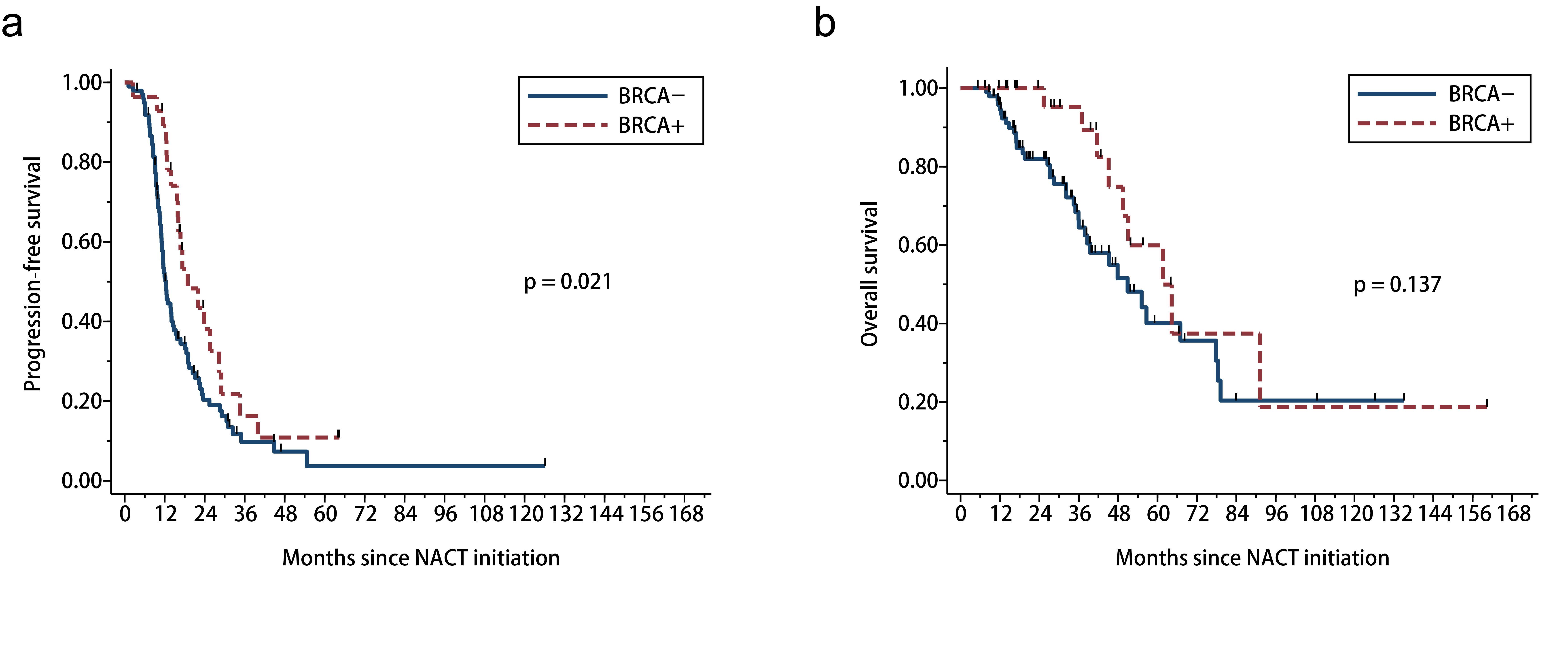

Supplement: Supplementary file 1 [file cancers-13-00704-s001.zip › fig s1_BRCA.jpg]

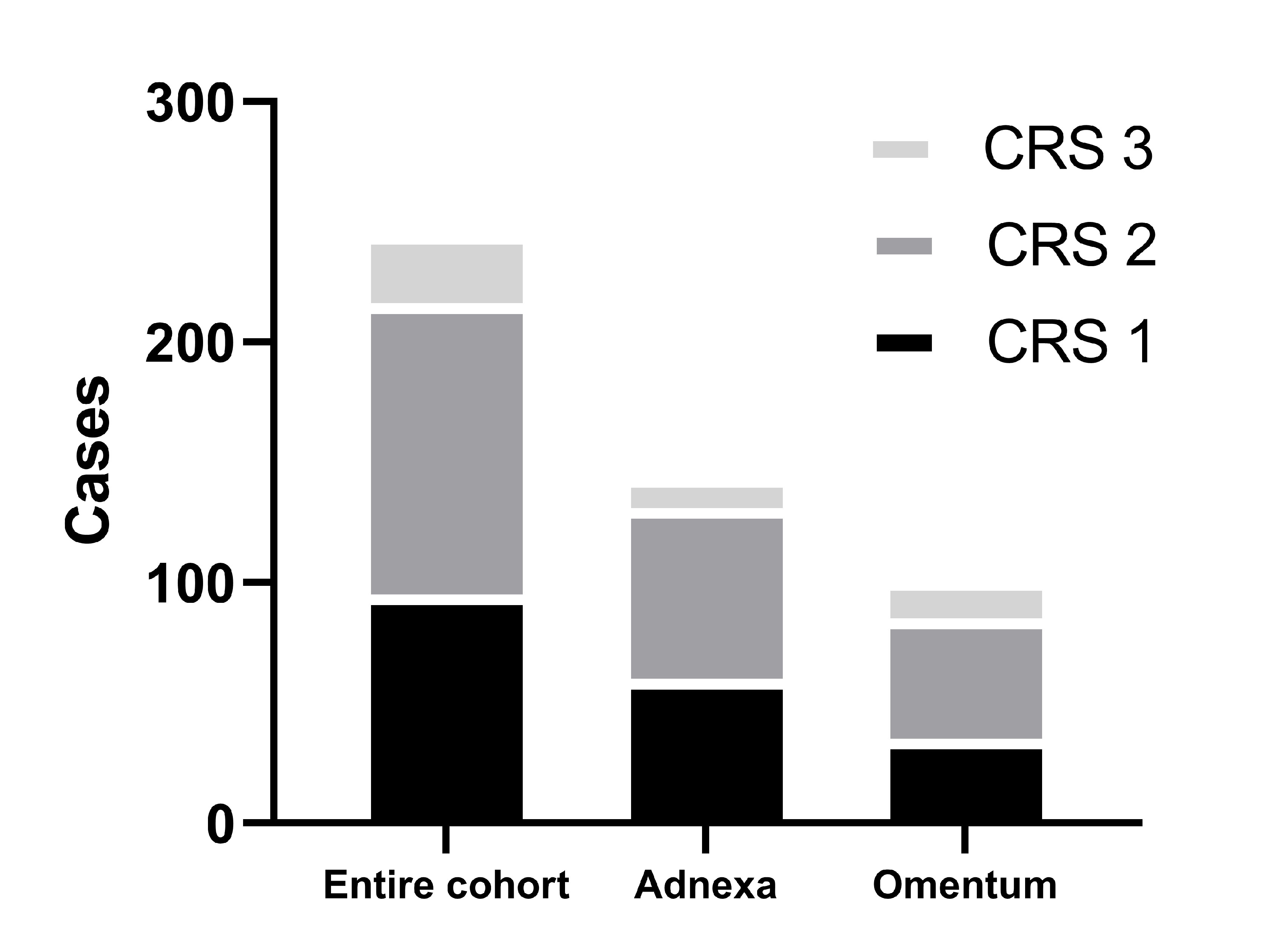

Supplement: Supplementary file 1 [file cancers-13-00704-s001.zip › fig s2_CRS distribution.jpg]
